# Targeting Orai1-Mediated Store-Operated Ca^2+^ Entry in Heart Failure

**DOI:** 10.3389/fcell.2020.586109

**Published:** 2020-10-08

**Authors:** Rui Luo, Ana-Maria Gomez, Jean-Pierre Benitah, Jessica Sabourin

**Affiliations:** Inserm, UMR-S 1180, Signalisation et Physiopathologie Cardiovasculaire, Université Paris-Saclay, Châtenay-Malabry, France

**Keywords:** orai1, store-operated Ca^2+^ entry, cardiomyocytes, heart, hypertrophy, heart failure

## Abstract

The archetypal store-operated Ca^2+^ channels (SOCs), Orai1, which are stimulated by the endo/sarcoplasmic reticulum (ER/SR) Ca^2+^ sensor stromal interaction molecule 1 (STIM1) upon Ca^2+^ store depletion is traditionally viewed as instrumental for the function of non-excitable cells. In the recent years, expression and function of Orai1 have gained recognition in excitable cardiomyocytes, albeit controversial. Even if its cardiac physiological role in adult is still elusive and needs to be clarified, Orai1 contribution in cardiac diseases such as cardiac hypertrophy and heart failure (HF) is increasingly recognized. The present review surveys our current arising knowledge on the new role of Orai1 channels in the heart and debates on its participation to cardiac hypertrophy and HF.

## Introduction

Spatiotemporal modulation of intracellular Ca^2+^ levels provides a signal transduction mechanism in virtually all cell types. This is used to determine which both short- and long-term cellular functions are activated and when. This fine control of Ca^2+^ handling is notably setup by various ion channels at the plasma membrane, including the store-operated Ca^2+^ channels (SOCs) and their corresponding store-operated Ca^2+^ entry (SOCE).

The notion of SOCE was first proposed in salivary gland cells as a “capacitative Ca^2+^ entry” (Putney, 1986, 1990), which couples the extracellular Ca^2+^ influx to the depletion of endoplasmic reticulum (ER) Ca^2+^ stores. Ca^2+^ influx into the cell *via* SOCs can be pumped back into the ER to replenish depleted stores and restore ER Ca^2+^ homeostasis.

After its initial study, direct evidence for the concept of a Ca^2+^ release-activated Ca^2+^ current (*I*_CRAC_) activated by Ca^2+^ store depletion in mast cells was provided by patch-clamp experiments (Hoth and Penner, 1992). *I*_CRAC_ was described as a sustained non-voltage-activated Ca^2+^ inward current with an inward rectification positive to reversal potential. It is highly selective for Ca^2+^ ions over Ba^2+^, Sr^2+^, and Mn^2+^ (Hoth and Penner, 1992). The modern molecular period identifies two essentials molecular candidates responsible for the SOCE and the *I*_CRAC_: one is the STIM1 protein, serving as an ER Ca^2+^ sensor *via* its N-terminal EF-hand domain; the second is Orai1 (also called CRACM1) forming the classical SOCs, which is highly selective for Ca^2+^. The participation of the transient receptor potential canonical (TRPCs), which are non-Ca^2+^ selective channels, as SOCs remains a highly contentious issue. The mechanism for SOCE activation was then elucidated by a series of elegant studies conducted primarily in non-excitable cells. Upon ER Ca^2+^ depletion, Ca^2+^ dissociates from the STIM1 EF-hand domain resulting in dimerization or oligomerization of STIM1 proteins and their translocation to the ER-plasma membrane (PM) junctions where they interact and activate Orai1 channels (Ma et al., 2015).

Orai1 is a ∼33-kDa protein composed of 301 amino acids, although the predicted molecular weight might be significantly modified by post-translational modifications, with four putative transmembrane-spanning domains (M1–M4) and cytosolic NH2- and COOH-tails (Rosado et al., 2015). M1 lines the ion conduction pathway of Orai1 and contains several amino acid residues that define the biophysical properties of the channel (Lacruz and Feske, 2015). A crystal structure of the *Drosophila melanogaster* Orai channel, which shares 73% sequence identity with human Orai1 within its transmembrane region, has been determined (Hou et al., 2012). This study revealed a hexameric assembly of Orai1 proteins around a central ion pore, which crosses the membrane and extends into the cytosol, with extracellular glutamate residues forming a selectivity filter. The hexameric quaternary assembly is in contrast with several previous studies suggesting that Orai1 assembles as a functional tetramer (Penna et al., 2008; Maruyama et al., 2009).

Orai1 exhibits a wide functional distribution across mammalian cells and tissue types and is essential for SOCE (Feske et al., 2006; Vig et al., 2006). Indeed, Orai1 as the pore-forming SOC unit was discovered throughout a genetic analysis of patients with autosomal recessive loss-of-function (LOF) or gain-of-function (GOF) mutations in *ORAI1*, which are associated with severe combined immunodeficiency (SCID)-like disease, tubular aggregate myopathy (TAM), and Stormorken syndrome (Feske, 2019). The revealed missense *ORAI1* mutation is associated with defective SOCE and *I*_CRAC_, resulting in loss of fine control of Ca^2+^-mediated processes characterized by a severe immunodeficiency with myopathy. Nowadays, there is emerging evidence that Orai1 channels are important to almost every cell type. In cardiomyocytes, their potential physiological and pathological roles during cardiac hypertrophy and HF processes have been intensively investigated in the past few years.

The present review will survey our current knowledge on the expression and function of Orai1 in the heart and on its participation to cardiac hypertrophy and HF.

## Physiological Role of Orai1 in the Heart

### Expression and Cellular Location

The presence of SOCE was first described in embryonic and neonatal rat ventricular cardiomyocytes (NRVMs) (Hunton et al., 2002; Uehara et al., 2002) and then in adult rat ventricular cardiomyocytes (Hunton et al., 2004). This was further confirmed by the expression of the molecular SOCs actors, such as Orai1 channels in the cardiomyocytes (Table 1).

**TABLE 1:** Orai1 expression in the heart.

**Interfering methods**	**Techniques**	**Expression level**	**Antibody**	**Species**	**Cell type/tissue**	**Proposed location**	**References**
MO-Orai1/siRNA	qRT-PCR	mRNA/Protein	Santa Cruz Biotech. (KD validated)	Zebrafish	One-cell embryos	PM	Volkers et al., 2012
	WB			Rat	Neonatal cardiomyocytes		
				Mouse	Embryonic hearts		
	IHC				Adult hearts		
	PLA				Isolated adult cardiomyocytes		
siRNA	qRT-PCR/WB/IC	mRNA/Protein	N/A (KD validated)	Rat	Neonatal cardiomyocytes	Perinuclear and cytosolic	Voelkers et al., 2010
RNAi	RT-PCR/WB	mRNA/Protein	Millipore	Mouse	HL-1 cell line		Touchberry et al., 2011
Cardiomyocyte-specific expression dn-Orai1^*R91W*^	qRT-PCR/WB/IC/IHC	mRNA/Protein	Sigma-Aldrich (08264) (Kl and KO validated)	Mouse	Adult Hearts/Isolated adult cardiomyocytes	PM/IDs/nuclei	Bartoli et al., 2020
	qRT-PCR/WB	mRNA/Protein	Sigma-Aldrich (08264)	Rat	Isolated adult cardiomyocytes		Bartoli et al., 2019
siRNA/dn-Orai1^G98A^ Myc-tagged Orai1/Myc-tagged Orai1^*S34A*^	qRT-PCR/WB/IC/IP	mRNA/Protein	Alomone (ACC-062) (KD validated)	Human	hESC-CMs/Neonatal cardiomyocytes	Puncta along the PM/nuclei	Wang et al., 2015
				Rat			
BTP2	qRT-PCR/WB/IC	mRNA/Protein	Sigma-Aldrich (08264)/ProSci (PM-5207)	Mouse	SAN tissue and cells/Atria/Ventricles tissue	PM	Liu et al., 2015
2-APB (as activator)	WB/IHC	Protein	ProSci (4281)/ProSci (4217)	Rat	Left atria tissue/Papillary muscle	Puncta	Wolkowicz et al., 2011
2-APB (as activator)	WB/IHC	Protein	ProSci (4281)/ProSci (4217)	Rat	Left ventricles tissue	Diffuse/Puncta	Wang et al., 2012
siRNA	qRT-PCR/WB/Co-IP	mRNA/Protein	Santa Cruz Biotech. (sc-68895)(KD validated)	Rat	Isolated adult cardiomyocytes		Saliba et al., 2015
siRNA	RT-PCR/WB/IC/Co-IP	mRNA/Protein	N/A (KD validated)	Human	Cardiac c-kit^+^ progenitor cells	PM	Che et al., 2015
dnOrai1^*E106A*^/S66/BTP2	qRT-PCR/WB/IC/Co-IP	mRNA/Protein	Sigma-Aldrich (08264)	Rat	Neonatal cardiomyocytes		Sabourin et al., 2016
Orai1^+/–^	WB	Protein		Mouse	Adult heart tissue		Horton et al., 2014
	WB	Protein		Rat	Isolated adult cardiomyocytes		Dominguez-Rodriguez et al., 2015
dn-Orai1^R93W^	qRT-PCR	mRNA		Mouse	Heart tissue		Maus et al., 2017
	qRT-PCR	mRNA		Chicken	Heart tissue		Li et al., 2017
BTP2/dn-Orai1^*E106Q*^	qRT-PCR/IC	mRNA/Protein		Cat	Isolated adult cardiomyocytes	PM	Troupes et al., 2017
GSK7579A/S66	WB/IC	Protein	Alomone (ALM-25)	Mouse	Heart tissue/Isolated adult cardiomyocytes	IDs	Bonilla et al., 2019
	WB	Protein	Millipore	Mouse	Ventricle tissue		Correll et al., 2015
	IC	Protein		Mouse	Isolated adult cardiomyocytes	Nucleoplasm	Lee et al., 2018
	WB/IC	Protein	Cell signaling (#1280)	Rat	Heart tissue/Neonatal cardiomyocytes	Membrane fraction Puncta along the PM	Zhu-Mauldin et al., 2012
	WB/Co-IP		Santa Cruz Biotech.		Heart tissue		Lu et al., 2017
	WB	Protein	ProSci (4281)	Mouse	Isolated adult cardiomyocytes		Collins et al., 2014
	WB/Co-IP	Protein	Abcam (sc-377281)	Rat	Neonatal cardiomyocytes		Malette et al., 2019
CRACM1^+/+^-LacZ	LacZ staining	Protein		Mouse	Neonatal heart		Vig et al., 2008
siRNA	WB	Protein	Sigma-Aldrich (08264) (KD validated)	Mouse	Heart tissue		Zheng et al., 2017
				Rat	Neonatal cardiomyocytes		
	qRT-PCR/WB/IHC	mRNA/Protein	Abcam	Mouse	Heart tissue/Neonatal cardiomyocytes		Dai et al., 2018
Orai1^Flox/Flox^/α-MHC/Cre	qRT-PCR/IC	mRNA/Protein	N.A. (#1003) (KO validated)	Mouse	Isolated embryonic cardiomyocytes Isolated adult cardiomyocytes		Segin et al., 2020
	qRT-PCR	mRNA		Human	Left ventricle tissue		Cendula et al., 2019
	IHC/IC	Protein	Alomone	Mouse	SAN/SANCs	PM	Zhang et al., 2015
	WB	Protein	Santa Cruz Biotech (sc-68895)	Mouse	HL-1 cell line		Shiou et al., 2019
	RT-PCR/WB/Co-IP	mRNA/Protein		Human	Atria tissue		Zhang et al., 2013
	qRT-PCR	mRNA		Ground squirrels	Papillary muscles		Nakipova et al., 2017
	Northern-blot	RNA		Mouse	Heart tissue		Gross et al., 2007
	WB	Protein	Abcam	Rat	Neonatal cardiomyocytes		Ji et al., 2017
	RT-PCR	mRNA		Mouse	Heart tissue		Takahashi et al., 2007
siRNA	PCR-based micro-array/WB/PLA	Protein	Novus Biologicals (KD validated)	Rat	Neonatal cardiomyocytes/Heart tissue		Dominguez-Rodriguez et al., 2018
	IHC	Protein	mAb266.1/ProSci (4281)	Rat	Heart tissue		Guzman et al., 2014
				Human			
	WB	Protein	Sigma-Aldrich (08264)	Rat	Left and right ventricles tissues		Sabourin et al., 2018

*WB, Western-blot; IC, immunocytochemistry; IHC, immunohistochemistry; PLA, proximity ligation assay; IP, immunoprecipitation; Dn, dominant negative; hESC-CMs, human embryonic stem cell-derived cardiomyocytes; PM, plasma membrane; IDs, intercalated disks.*

Expression of Orai1 protein was first detected in 1-day-old neonate mice heart (Vig et al., 2008). Later on, many other studies described its expression in the NRVMs (Voelkers et al., 2010, Volkers et al., 2012; Zhu-Mauldin et al., 2012; Wang et al., 2015; Sabourin et al., 2016; Ji et al., 2017; Zheng et al., 2017; Dominguez-Rodriguez et al., 2018; Dai et al., 2018; Malette et al., 2019). Orai1 expression decreased after birth in mice (Volkers et al., 2012). In the adult heart, ventricular tissues, and isolated ventricular cardiomyocytes from rats, mice, cats, chickens, zebrafish, or even hibernator animal squirrels, Orai1 was also detected with a low or even moderate level of expression in rodents (Gross et al., 2007; Takahashi et al., 2007; Volkers et al., 2012; Wang et al., 2012; Zhu-Mauldin et al., 2012; Collins et al., 2014; Guzman et al., 2014; Horton et al., 2014; Correll et al., 2015; Dominguez-Rodriguez et al., 2015; Liu et al., 2015; Saliba et al., 2015; Li et al., 2017; Lu et al., 2017; Maus et al., 2017; Nakipova et al., 2017; Troupes et al., 2017; Zheng et al., 2017; Dai et al., 2018; Dominguez-Rodriguez et al., 2018; Lee et al., 2018; Sabourin et al., 2018; Bartoli et al., 2019; Bonilla et al., 2019; Segin et al., 2020). Orai1 is also expressed in HL-1 atrial muscle cell line (Touchberry et al., 2011; Shiou et al., 2019), in left atrial rat and mice myocytes (Wolkowicz et al., 2011; Liu et al., 2015), in human embryonic stem cell-derived cardiomyocytes (Che et al., 2015; Wang et al., 2015), and in the sarcolemma of mouse sinoatrial node cells (SANCs) and tissue (Zhang et al., 2013; Liu et al., 2015).

Of note, one research group could not detect the Orai1 expression in isolated adult rat cardiomyocytes (Zhang et al., 2015). This might be related not only to its weak expression but also to the antibody used.

Orai1 expression is more abundant in human myocardial tissue (Guzman et al., 2014), in human left ventricular tissue (Cendula et al., 2019), and in human atrial myocytes (Zhang et al., 2013) than in rodents.

Limited numbers of studies have investigated the cellular distribution of Orai1. The Orai1 location seems somewhat inconsistent depending on the cell types studied, in particular between neonatal and adult cardiomyocytes. This is probably due to the different techniques for cell fixation and permeabilization and/or the antibody specificities.

In NRVMs, a surprising perinuclear and cytosolic pattern for Orai1 has been reported (Voelkers et al., 2010). In NRVMs overexpressing Orai1, upon a passive Ca^2+^ SR depletion, Orai1 was redistributed as puncta in the SR-PM junctions, with STIM1 promoting their interaction (Zhu-Mauldin et al., 2012). Orai1 and STIM1 were also found in the form of aggregates at the peripheral membrane of cardiomyocytes derived from human pluripotent stem cells (hESC-CMs) in the absence of SR Ca^2+^ depletion (Wang et al., 2015).

In isolated adult ventricular cardiomyocytes from rats and mice, Orai1 has been located at the surface sarcolemma, with higher concentration at the intercalated disks (IDs) (Volkers et al., 2012; Bonilla et al., 2019; Bartoli et al., 2020). Furthermore, it has been shown that the STIM1/Orai1 complexes were enriched at N-cadherin-rich IDs sites over Cx43-rich sites in adult mice cardiomyocytes (Bonilla et al., 2019). We also noted occasional nuclear Orai1 labeling in isolated mice cardiomyocytes (Bartoli et al., 2020). Indeed, the location of Orai1/STIM1 complex in the nucleoplasm has been reported by other authors as well (Lee et al., 2018), suggesting Orai1 involvement in nucleoplasmic Ca^2+^ regulation. In isolated pacemaker cells, Orai1 was colocalized with the hyperpolarization-activated cyclic nucleotide-gated (HCN)4 channels at the surface membrane (Liu et al., 2015; Zhang et al., 2015).

### Orai1 Function

Overall, most of the reports have detected Orai1 expression in cardiomyocytes. However, the physiological role of the Orai1-mediated Ca^2+^ entry into the heart remains somewhat unclear and enigmatic. As for other excitable cells, it has long been considered that SOCE contribution to cardiac Ca^2+^ homeostasis is rather limited or non-existent, taking into account that Ca^2+^ influx during each beat through voltage-dependent Ca^2+^ channels is large enough to maintain cardiac excitation-contraction coupling (ECC). However, even a modest change in Ca^2+^ signaling can progressively alter heart function if sustained. For a prolonged period, a source of Ca^2+^ through SOCs can therefore potentially affect the heart.

The Orai1 knockdown (KD) in NRVMs did not affect the diastolic Ca^2+^ level or the SR Ca^2+^ load (Voelkers et al., 2010). However, the spontaneous Ca^2+^ transients frequency was reduced, as well as the NRVMs size. This was associated with the reduced activity of Ca^2+^-sensing proteins, such as calcineurin (CaN), calmodulin-dependant kinase II (CaMKII), or extracellular signal-regulated kinases (ERK1/2), signaling pathways involved in cardiac hypertrophy, and HF. Conversely, the reduction of SOCE in the HL-1 cell line with Orai1 KD decreased the diastolic Ca^2+^ level and SR Ca^2+^ content, suggesting a role of Orai1 in the SR Ca^2+^ load maintenance (Touchberry et al., 2011). More recently, we have demonstrated that the mineralocorticoid receptor pathway promotes an Orai1-dependent SOCE, which regulated the diastolic Ca^2+^ level *via* the serum and glucocorticoid-regulated kinase 1 (SGK1) in NRVMs (Sabourin et al., 2016). In hESC-CMs, the functional inhibition of Orai1 using overexpression of a dominant negative Orai1^G98A^ mutant or the KD with shRNA against Orai1 decreased the cell size and altered the sarcomere organization (Wang et al., 2015). In human cardiac c-kit^+^ progenitor cells, Orai1-mediated SOCE regulated cell cycling and migration *via* cell cycle kinase cyclin D1 and cyclin E and/or phosphorylation of Akt (Che et al., 2015).

The role of Orai1 in maintaining cytosolic and intrareticular Ca^2+^ levels during development therefore raises the question of its potential involvement in the adult heart.

A recent study, in adult mouse cardiomyocytes, has identified highly localized SOCE events preferentially at the IDs, where STIM1-Orai1 complex formation occurred in close proximity to intracellular mechanical junctions (Bonilla et al., 2019). This microdomain segregation might participate in arrhythmogenesis without interfering in the ECC process. This echoed that, in adult feline cardiomyocytes, the BTP2, a classic Orai1 inhibitor, did not modify the action potential duration (APD), neither Ca^2+^ transients nor cell contraction (Troupes et al., 2017).

*In vivo*, antisense oligonucleotide strategy to knockdown Orai1 in zebrafish leads to spontaneous ventricular systolic dysfunction and bradycardia, reduced blood circulation, blood congestion without affecting cardiogenesis and cardiomyocyte differentiation (Volkers et al., 2012). This was associated with ultrastructure alterations of sarcomeres, leading to reduced expression and impaired z-disc localization of calsarcin involved in the activation of calcineurin/nuclear factor of activated T cell (CaN/NFAT) hypertrophic pathway. In addition, inducible RNAi to specifically suppress Orai1 expression in the *Drosophila* heart resulted in significant delays in post-embryonic development, premature death in adults, and impaired cardiac function due to myofibril disorganization consistent with dilated cardiomyopathy (Petersen et al., 2020).

In contrast, heterozygous global Orai1-deficient mice did not present alteration of heart structure and function (Horton et al., 2014). Likewise, we have reported that cardiomyocyte-specific dn-Orai1^R91W^ transgenic mice displayed normal cardiac electromechanical function and ECC despite reduced Orai1-dependent SOCE (Bartoli et al., 2020), as recently confirmed in cardiomyocyte-specific and temporally inducible Orai1 knockout (KO) mouse line (Segin et al., 2020). Although in the later study, this was associated with reduced body weight and a downregulation of Orai3, STIM2, and store-operated Ca^2+^ entry-associated regulatory factor (SARAF) transcript expression, those results suggested that Orai1 is not instrumental for the ECC and cardiac function at rest. In addition, it has been shown that dn-Orai1^R93W^ mice display abnormal amounts of lipid droplets in the heart, providing a potential role in lipid metabolism (Maus et al., 2017).

In pacemaker cells from mouse, SOCE inhibition by BTP2 reduced the frequency and the amplitude of spontaneous Ca^2+^ transients and the SR Ca^2+^ content (Liu et al., 2015). It has been speculated that STIM1/Orai1-dependent SOCE were activated by the rhythmic release of Ca^2+^ from the SR, which activates Orai1 to refill the SR stores. In this way, STIM1 and Orai1 ensured the fidelity of SAN Ca^2+^ dynamics and the integrity of the cardiac pacemaker (Zhang et al., 2015).

Two studies have linked the non-selective Orai1 activator, 2-aminoethoxydiphenyl borate (2-APB at 20 μM) to the initiation of atrial and ventricular arrhythmias (Wolkowicz et al., 2011; Wang et al., 2012). Indeed, 2-APB induced sporadic or tachycardial ectopic activities, as well as automatic activity in the superfused left rat atrium and left rat ventricular papillary muscles. The common non-specific SOC inhibitor, the SKF-96365, suppressed those arrhythmic behaviors (Wolkowicz et al., 2011). In addition, on a model of isolated rat hearts perfused by the Langendorff method, 2-APB also promoted ventricular fibrillations, which were prevented by SKF-96365. These studies suggest that Orai1 may be an important regulator of the electrical stability in rat hearts (Wang et al., 2012).

Taken together, it is generally accepted that SOCE carried by Orai1 channels regulates the SR Ca^2+^ content, the diastolic Ca^2+^, as well as cell growth during cardiac development. However, in adulthood, additional studies are clearly necessary to clarify the controversy over the role of SOCE machinery in the heart.

## Pathophysiological Role of Orai1 in Cardiac Hypertrophy and Heart Failure

Whether Orai1 role in heart physiology is still elusive, its involvement in pathophysiological situation has been more documented.

As terminally differentiated cells, adult cardiomyocytes are largely incapable of cell proliferation. Hypertrophic growth from different pathologic stimuli, including hypertension, coronary insufficiency, or valvular defects, is the primary adaptive mechanism by which the heart is able to preserve pump function and maintain adequate cardiovascular support. On the other hand, sustained hypertrophy can lead to altering myocardial architecture and to cardiac dysfunction, dilated cardiomyopathy, HF, and sudden death. The dysregulation of cardiac Ca^2+^ homeostasis is an important and proximal player underlying the pathogenesis of heart diseases. As the main regulator of the cardiac ECC, mishandling of Ca^2+^ is directly related to the mechanical dysfunction and certain arrhythmias associated with hypertrophy and HF. Moreover, the Ca^2+^-dependent intracellular signaling pathways activation, notably CaN/NFAT, CaMKII, and ERK promotes pro-hypertrophic gene expression, leading to pathological growth, cardiac remodeling, and dysfunction (Wilkins et al., 2004; Mattiazzi et al., 2015; Gallo et al., 2019). However, the sources of Ca^2+^ responsible for the activation of these Ca^2+^-dependent signaling circuits are still elusive. Nonetheless, in the last 20 years, convincing evidences have proposed a key role for Orai1-mediated SOCE in such processes.

### Orai1 Expression During Hypertrophic Process

Several studies have found that the expression of Orai1 was enhanced in angiotensin II (AngII) or phenylephrine (PE)-induced hypertrophy of NRVMs or hESC-CMs (Wang et al., 2015; Ji et al., 2017; Zheng et al., 2017; Dai et al., 2018). Increased Orai1 expression was also observed after ischemia/reperfusion in NRVMs (Dominguez-Rodriguez et al., 2018). Several *in vivo* studies also found Orai1 mRNA and protein expression upregulation in mouse cardiac hypertrophic model induced by pressure and volume overload, such as transverse aortic constriction, abdominal aortic banding, chronic AngII infusion, or myocardial infarction (Volkers et al., 2012; Dai et al., 2018; Bartoli et al., 2020; Segin et al., 2020). In right ventricular hypertrophy and dysfunction secondary to pulmonary hypertension, we also observed an increased Orai1 expression (Sabourin et al., 2018). Surprisingly, Orai1 expression was decreased by 30% in the end-stage human failing left myocardium (Cendula et al., 2019). Interestingly, this decrease was gender specific, present only in men, suggesting that Orai1 expression might represent a possible mechanism of cardioprotective effects of estrogens (Cendula et al., 2019). By contrast, the fibroblasts from end-stage human left failing patients have increased collagen secretion capacity, which was related to increased SOCE and enhanced expression of Orai1 (Ross et al., 2017).

### *In vitro* Functional Studies

In NRVMs, non-selective inhibitors of SOCs, such as glucosamine or SKF-96365, prevented the increase of NFAT nuclear translocation and cellular hypertrophy after a 48-h treatment with hypertrophic stressors (AngII or PE) (Hunton et al., 2002). Furthermore, in the same model, SOCE inhibition by R02959 or by Orai1 KD prevented pro-hypertrophic signaling such as the nuclear localization of NFAT, the Gq-protein conveyed activation of the CaMKII/ERK1/2 signaling pathway, and CaN activation (Voelkers et al., 2010; Zheng et al., 2017). In PE-induced hypertrophy of NRVMs, in one hand, CaMKIIδ inhibition by siRNA or by KN93 normalized the Orai1 protein level, as well as the hypertrophic marker. On the other hand, BTP2 treatment attenuated the hypertrophic growth and the increased CaMKIIδ expression (Ji et al., 2017). The CaMKIIδ upregulation might thus contribute to the PE-induced cellular hypertrophy through the overactivation of SOCE and/or conversely the enhanced SOCE induced by PE led to overexpression and activation of CaMKIIδ in the cardiomyocytes.

In a mouse model and cultured cardiomyocyte model treated with AngII or PE, gastrodine, a polyphenol with anti-inflammatory properties used in traditional Chinese medicine, is protective against the development of cellular and cardiac hypertrophy by attenuating the SOCE and reducing the expression of STIM1 and Orai1 (Zheng et al., 2017).

In hECS-CMs, Orai1 inhibition by the use of Orai1-siRNAs or a dominant-negative construct Orai1^G98A^ or by nitric oxide (NO) *via* activation of PKG prevented PE-induced cellular hypertrophy (Wang et al., 2015). Of note, the anti-hypertrophic effects of NO, cGMP, and PKG were lost when Orai1 was mutated on serine 34. Indeed, Ser34 can be phosphorylated by PKG, which decreases Orai1-mediated SOCE and therefore inhibits the development of cellular hypertrophy. These results provided novel mechanistic insights into the action of cGMP-PKG-related anti-hypertrophic agents, such as Sildenafil. More recently, AngII-induced cellular hypertrophy was blunted in embryonic cardiomyocytes from cardiomyocyte-specific Orai1-KO mice (Segin et al., 2020).

Of note, the pharmacology of Orai1 channels is poor and lack of specificity. Indeed, 2-APB was originally described as an inhibitor of the IP_3_R. BTP2 was known to inhibit TRPC channels and activate TRPM4. SKF-96365 has been shown to block voltage-gated Ca^2+^ channels, K^+^ channels, and TRP family members (Prakriya and Lewis, 2015; Bird and Putney, 2018). To avoid misinterpretation of the pathophysiological role of Orai1, it is thus essential to use KD or KO strategies in addition to the pharmacological tools.

All of these *in vitro* studies demonstrate the involvement of Orai1-mediated SOCE in hypertrophic development induced by neurohormonal stimulation. The neurohormonal-induced hypertrophy pathways imply an increase in the activity of pro-hypertrophic signaling such as CaN/NFAT, CaMKII, and ERK1/2 *via* enhanced Orai1 expression and activity.

### *In vivo* Functional Studies

In pressure overload-induced cardiac adaptive hypertrophy in mice, overexpression of SARAF in the heart prevented Orai1 upregulation and attenuated the cardiac hypertrophy. In addition, overexpression of SARAF also attenuated AngII-induced upregulation of Orai1 and hypertrophy of cultured cardiomyocytes (Dai et al., 2018). In similar pathological model in cats, BTP2 did not prevent the Ca^2+^ alterations observed in enlarged adult feline cardiomyocytes, namely the decrease in the amplitude and the prolongation of Ca^2+^ transients and the prolongation of contraction. However, it prevented the shortening of sarcomeres in diastole; the increase in the APD as well as the Ca^2+^ sparks frequency (Troupes et al., 2017). Overexpression of STIM1 in cultured adult feline ventricular myocytes also increased the SR Ca^2+^ load, the diastolic spark rate, and prolonged APD and activated CaMKII. STIM1 effects were eliminated by either BTP2 or by coexpression of a dominant negative Orai^E106Q^ mutant (Troupes et al., 2017). These results supported the idea that, during hypertrophic stress, STIM1/Orai1 produced an exacerbated Ca^2+^ influx that can prolong the APD and load sufficiently the SR for each cycle of contraction. However, in the long term, it can induce Ca^2+^ leak from the SR through an alteration of the CaMKII-dependent phosphorylation state of RyR and likely contributed to the altered electromechanical properties of the hypertrophied heart (Troupes et al., 2017).

Long-term administration of pyridostigmine, an acetylcholinesterase inhibitor, alleviated pressure overload-induced cardiac hypertrophy as well as associated fibrosis (Lu et al., 2017). This beneficial effect was related to the inhibition of the CaN/NFAT3/GATA4 pathway and suppression of the interaction of Orai1/STIM1.

We also found that after chronic pressure overload, cardiomyocyte-specific dn-Orai1^R91W^ mice (C-dnO1), or *in vivo* JPIII-treated (a new selective Orai1 inhibitor) mice were protected from left ventricular systolic dysfunction and from interstitial fibrosis deposition, even if increased cardiac hypertrophy was observed. This was correlated with a protection from pressure overload-induced cellular Ca^2+^ signaling alterations (increased SOCE, decreased [Ca^2+^]_i_ transients amplitude and decay rate, lower SR Ca^2+^ load, and depressed cellular contractility), SERCA2a downregulation, and Pyk2/MEK/ERK overactivation (Bartoli et al., 2020). We observed an increased CRAC-like current, associated with longer APD, higher and faster [Ca^2+^]_i_ transients, and increased SR Ca^2+^ content and cell contractility in the right ventricular hypertrophy secondary to pulmonary hypertension (Sabourin et al., 2018). Pharmacological inhibition of Orai1 channels by BTP2 in hypertrophied RV cardiomyocytes normalized the [Ca^2+^]_i_ transients amplitude, the SR Ca^2+^ content and cell contractility to control levels (Sabourin et al., 2018). These new findings demonstrated that the Orai1-dependent Ca^2+^ current participated in cardiac Ca^2+^ remodeling in the right ventricular hypertrophy secondary to pulmonary hypertension.

All these studies seem contradictory with the results obtained with global heterozygous Orai1^+/–^ (Horton et al., 2014). These mice subjected to pressure overload have reduced survival and develop more rapid dilated cardiomyopathy with greater loss of function than control littermates. The loss of Orai1 thus accelerated the development of dilated cardiomyopathy and HF. The cardiac hypertrophy level was similar, however, Orai1^+/–^ mice were no longer able to compensate the chronic pressure overload, resulting in the development of a more severe systolic dysfunction. This can be explained by significant apoptosis without differences in hypertrophic and fibrotic markers, although this was not clearly demonstrated (Horton et al., 2014). Similarly, cardiomyocyte-specific deletion of Orai1 in adult mice (Orai1CM-KO) did not protect them from AngII-induced cardiac dysfunction (Segin et al., 2020). Despite disparity in echocardiographic data, Orai1CM-KO mice presented a slight decrease in systolic function with an accumulated fibrosis compared with control mice suggesting a transition to a maladaptive hypertrophy.

Despite discrepancies between all the *in vitro* and *in vivo* studies certainly due to the different biomechanical stresses and the cardiac phenotype induced, all the published results so far support the idea that Orai1 is a critical mediator for Ca^2+^ entry and cardiac remodeling.

## Pharmacological Opportunities of Orai1 in Cardiac Diseases

HF, the end stage of almost all heart diseases, is a major health problem in the world today, affecting over 26 million persons worldwide with increased prevalence. Current treatment is mainly correlated with medications, diet, interventions, devices, and heart transplants (Seferovic et al., 2019). Whereas the outcome of HF has improved significantly in the last quarter century, the mortality still remains high, with nearly half of the patients with HF dying within 5 years after diagnosis.

Current pharmacological therapies for HF with reduced ejection fraction are largely either repurposed anti-hypertensives that blunt overactivation of the neurohormonal system or diuretics that decrease congestion. They do not address the decrease in cardiac systolic function, a central factor in HF that results in reduction in cardiac output and reserve. Numerous attempts have been made to develop and test positive cardiac inotropes that improve cardiac hemodynamics, but adverse effects inherent to their mechanism of benefit limit their uses. Intravenous positive inotropic drugs including phosphodiesterase (PDE)-3 inhibitors (e.g., milrinone), β-adrenergic receptor agonists (e.g., dobutamine), Ca^2+^-sensitizing agents (e.g., levosimendan), and digoxin are indicated for acute systolic HF patients with decreased cardiac contractility and evidence of end-organ hypoperfusion (Tariq and Aronow, 2015). However, the use of positive inotropic drugs has been plagued by serious concerns regarding increased morbidity and mortality. Problems include increased atrial or ventricular arrhythmias, induced myocardial ischemia, and in some cases, arterial hypotension (Ahmad et al., 2019). Moreover, long-term use of conventional inotropic agents has been associated with no improvement in overall mortality (Ahmad et al., 2019). There is a possibility that the adverse effects of inotropic agents could promote pump failure as well as arrhythmias *via* dysfunctional Ca^2+^ cycling.

This uncomfortable dilemma has led to expand new and far more powerful methods of preventing and treating HF. Currently, HF drug treatment research focuses on interrupting intracellular signaling pathways that are injurious to cardiovascular tissue and on stimulating signaling pathways that protect cardiovascular tissues.

There is an arsenal of pharmacological selective inhibitors that modulate Orai1 function, and several have now advanced into human clinical trials for psoriasis, pancreatitis, asthma, or Hodgkins lymphoma (Stauderman, 2018). Consequently, there has been considerable interest in identifying Orai1 modulators, excluding immune diseases, as therapeutics for more conditions.

Over the past few years, as described herein, STIM1/Orai1-mediated SOCE has emerged as a promising target to treat HF. Here arises a question of which STIM1 and/or Orai1 should be targeted since both molecules operate in the same pathway. From a safety or toxicological perspective, Orai1 may be a more attractive target than STIM1. Firstly, compared with STIM1, Orai1 appears to be more restricted in its function of mediating SOCE. STIM1 appears to be involved in the activity of other proteins than Orai1, such as Cav1.2 (Wang et al., 2010; Dionisio et al., 2015) and TRPC channels (Yuan et al., 2007; Bodnar et al., 2017). STIM1 is also connected to the universal cAMP/PKA signaling pathway by regulating plasma membrane adenylate cyclases isoforms (Lefkimmiatis et al., 2009; Spirli et al., 2017; Motiani et al., 2018). As a result, drugs targeting STIM1 might lead to a poor benefit-to-risk ratio. Secondly, as exemplified in channelopathy caused by GOF mutations in Orai1 resulting in constitutive or increased SOCE independent of STIM1 (Lacruz and Feske, 2015), therapeutic targeting of STIM1 might not be as efficient, taking into account that reduction of STIM1 might accelerate transition to HF (Benard et al., 2016). Taken together, pharmacological targeting on Orai1 may produce less side effects.

According to the study conducted in our lab (Bartoli et al., 2020), under physiological conditions, cardiomyocyte-specific functional inhibition of Orai1 channels in C-dnO1 mice or systemic *in vivo* pharmacological Orai1 small-molecule inhibition by JPIII have little impact on Ca^2+^ homeostasis related to ECC and left ventricular systolic performance. After chronic pressure overload, JPIII markedly improves the left ventricular systolic function and Ca^2+^ handling by preventing the Ca^2+^ cycling mishandling, SERCA2a downregulation and fibrosis, without causing adverse effect. Our findings suggest that Orai1 inhibition has a potential favorable hemodynamic value to protect the heart from maladaptive hypertrophy and might represent a new inotropic support to help to relieve systolic dysfunction. This plausible therapeutic intervention need to be extended to other pathological models (Benitah et al., 2020). In addition, since Orai1 regulated cellular function in many tissues, concerns might raise about the safety and/or toxicity of Orai1 inhibitor due to potential chronic immunosuppression. In humans, the lack of function of Orai1 is dominated clinically by immunodeficiency with mostly normal overall T, B, and NK cell counts. In pilot studies, we have demonstrated that systemic chronic JPIII infusion for 28 days in mice did not induce any adverse effects, did not compromise the immune system, and did not promote susceptibility to develop infections (Bartoli et al., 2020). Currently, no adverse effect has been reported in clinical trials with selective Orai1 inhibitors such as AnCoA4, CM2489, CM4620, and Auxora (Stauderman, 2018; Miller et al., 2020). Nonetheless, given the important functional role of Orai1 in the immune system, it is important to properly understand the risks of potential adverse effects for a therapeutic approach moving forward.

In conclusion, although the Orai1 function in adult cardiac physiology remains elusive, converged experimental results pointed out a detrimental effect of Orai1 upregulation in hypertrophy and HF, including fibrosis, ventricular contractility, and pro-hypertrophic Ca^2+^-responsive signaling pathways. This pathological importance of Orai1 echoed its role during the embryonic and neonatal phase of cardiac development since hypertrophic cardiac remodeling is characterized by reactivation of the fetal gene program. Hence, Orai1-mediated SOCE may be a novel therapeutic target to consider in heart disease.

## Author Contributions

RL drafted the manuscript under the supervision of J-PB and JS. J-PB, JS, and A-MG edited the manuscript. All authors contributed to the article and approved the submitted version.

## Conflict of Interest

The authors declare that the research was conducted in the absence of any commercial or financial relationships that could be construed as a potential conflict of interest.
